# Advanced Computational Analysis of Cobalt-Based Superalloys through Crystal Plasticity

**DOI:** 10.3390/ma17102458

**Published:** 2024-05-20

**Authors:** Shahriyar Keshavarz, Carelyn E. Campbell, Andrew C. E. Reid

**Affiliations:** Thermodynamics and Kinetics Group, Material Measurement Laboratory, National Institute of Standards and Technology, Gaithersburg, MD 20899, USA; carelyn.campbell@nist.gov (C.E.C.); andrew.reid@nist.gov (A.C.E.R.)

**Keywords:** cobalt-based superalloys, composition, morphology, crystal plasticity, mechanical properties

## Abstract

This study introduces an advanced computational method aimed at accelerating continuum-scale processes using crystal plasticity approaches to predict mechanical responses in cobalt-based superalloys. The framework integrates two levels, namely, sub-grain and homogenized, at the meso-scale through crystal plasticity finite element (CPFE) platforms. The model is applicable across a temperature range from room temperature up to 900 °C, accommodating various dislocation mechanisms in the microstructure. The sub-grain level explicitly incorporates precipitates and employs a dislocation density-based constitutive model that is size-dependent. In contrast, the homogenized level utilizes an activation energy-based constitutive model, implicitly representing the $\gamma^{\prime }$ phase for efficiency in computations. This level considers the effects of composition and morphology on mechanical properties, demonstrating the potential for cobalt-based superalloys to rival nickel-based superalloys. The study aims to investigate the impacts of elements including tungsten, tantalum, titanium, and chromium through the homogenized constitutive model. The model accounts for the locking mechanism to address the cross-slip of screw dislocations at lower temperatures as well as the glide and climb mechanism to simulate diffusions at higher temperatures. The model’s validity is established across diverse compositions and morphologies, as well as various temperatures, through comparison with experimental data. This advanced computational framework not only enables accurate predictions of mechanical responses in cobalt-based superalloys across a wide temperature range, but also provides valuable insights into the design and optimization of these materials for high-temperature applications.

## 1. Introduction

For over half a century, nickel-based superalloys have stood as the preferred materials in the aerospace industry, particularly in the high-temperature zones of turbine engine components, such as blades and disks [1]. The exceptional mechanical properties, including remarkable yield stress and creep resistance at elevated temperatures, can be attributed to the distinctive γ-$\gamma^{\prime }$ microstructure characteristics [2]. Presently, nickel-based superalloys find application not only in aircraft engines, but also in power generation systems, where operating temperatures can reach up to approximately 1100 °C. The continuous evolution of nickel-based superalloys has been reached in compositions closely approaching their melting temperatures, prompting the need for the development of new high-temperature alloys [3]. The recent identification of a stable ternary system featuring an L12 phase in the Co–Al–W ternary by Sato et al. [4], capable of coexisting with an FCC solid-solution cobalt-based system, has opened new avenues for creating higher-temperature resistant materials in gas turbine applications. The γ-matrix phase is predominantly an alloy of Co having a face-centered cubic (FCC) lattice structure, while the precipitates or $\gamma^{\prime }$ phase is a coherent, ordered inter-metallic of an L12 crystal structure. The revelation of γ-$\gamma^{\prime }$ microstructure compositions akin to those in nickel-based superalloys [5] has sparked considerable research efforts [3,5] towards establishing a novel class of high-temperature, high-strength materials.

Considerable investments have been made to boost the efficiency of turbine engines, resulting in substantial demands for materials employed in their hot sections. The prevailing trend leans towards materials with higher melting points and extended service lifetimes, necessitating enhanced mechanical properties, particularly in terms of creep response. This is especially crucial in turbine blades, where temperatures approach approximately 90% of the material’s melting temperature [6]. In response to these advancements, computational approaches have been devised to accommodate these alterations through minor compositional adjustments. These approaches aim to explore the impacts of various elements such as W, Ta, Ti, Cr, etc., each with different concentrations, where empirical testing is conducted to assess their effects on crucial mechanical properties, including yield stress, plastic deformations, and creep properties. This is predominantly attributed to the intricate dislocation mechanisms and stress states prevalent in the hot sections, specifically in turbine engine components like blades and disks. Addressing these complexities necessitates the utilization of precise, physics-based models coupled with detailed constitutive models. This combination is essential for the development of robust computational methods, facilitating efficient analysis and design within a shorter time frame. These models must strike a balance between appropriate fidelity and practicality in terms of computational time and resources, given the intricacies involved. The fundamental dislocation mechanisms are heavily dependent on the microstructure’s morphology, where precipitates serve as hindrances to the continuous movement of dislocations. The dislocations either loop around or shear the precipitates, and the specific outcomes depend on factors such as temperature and stress level.

Creep tests on single crystals of cobalt-based superalloys have been conducted, with a focus on both cobalt (Co)-based and cobalt–nickel (CoNi)-based alloys [7,8]. The creep resistance observed in these alloys is comparable to that of single crystals belonging to the first-generation nickel-based superalloys. These first-generation alloys are characterized by a substantial volume fraction of 70% for cubic precipitates. While there have been significant studies on nickel-based superalloys, both experimentally and computationally, at low and high temperatures, and dislocation mechanisms, experimental data on high-temperature deformation mechanisms in cobalt-based superalloys are limited, and the corresponding computational methods are even less investigated.

Comprehensive analyses of the deformation behavior of nickel-based superalloys under various loading and temperature conditions have been conducted, especially for single crystals [9,10] and polycrystals [11,12,13]. Cobalt-based superalloys have been reported to exhibit similar deformation processes [14]. When the temperature varies, the dislocation processes in both scenarios changes. Octahedral slip systems are claimed to be activated in both phases at lower temperatures, and mechanical property anomalies are seen as the temperature increases.

In the current study, a comprehensive computational method using a crystal plasticity finite element (CPFE) approach is developed to explore the potential mechanical properties of cobalt-based superalloys. In doing so, several elements of Ta, Ti, and Cr are added to the ternary system of Co–Al–W to investigate the effects on the 0.2% flow stress as well as creep deformations. The computational framework needs to balance fidelity and performance at the same time, making multi-scale approaches a suitable choice. Multi-scale modeling techniques are effective computational tools whenever a problem of interest cannot be modeled on a single scale. The two-phase cobalt-based superalloys are one example where the average size of precipitates is much smaller than the microstructure’s dimensions. It is, as of now, computationally almost impossible to simulate the microstructure in the presence of all precipitates. Hence, multi-scale modeling techniques should be utilized in order to bridge the scales [15,16,17,18,19]. Homogenization theory, one of the techniques used in multi-scale modeling, is employed to develop constitutive models, including parametric forms of microstructural variables and their evolution [20,21]. The parametric forms and their evolution can accurately represent the effects of microstructural morphology and deformation mechanisms [22]. Meso-scale analyses of superalloys, incorporating precipitate distributions as well as grain structures, have been conducted using phenomenological viscoplastic constitutive laws in [23,24,25]. Hardening parameters in many of the constitutive models have been expressed as assumed functions of the average precipitate size.

The proposed multi-scale scheme is in the process of being implemented into the Object-Oriented Finite Element platform, OOF, developed by the scientists at the National Institute of Standards and Technology (NIST) [26], a publicly accessible platform intended to assist materials scientists and engineers in undertaking computational investigations of structure–property relations in a large variety of systems, including mechanical systems, whose behavior is dominated by crystal plasticity.

## 2. Multi-Scale Framework

**Figure 1:**
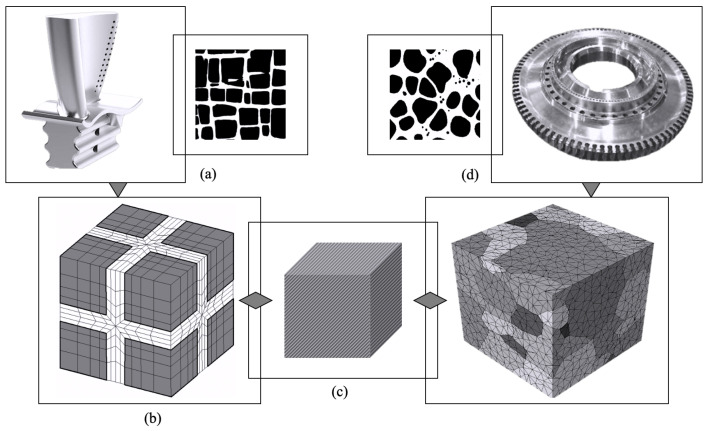
Multi-scale framework from single crystal to polycrystalline microstructures of superalloys: (**a**) Turbine blades with single crystalline microstructure and cubic precipitates. (**b**) Discretized finite element mesh of a single crystal description with a two-phase material. (**c**) Homogenized single crystal grain-scale finite element model. (**d**) Turbine disk with polycrystalline mictostructure.

The multi-scale method offers a substantial efficiency advantage, especially in simulating polycrystals, as there is no longer a need to consider explicit representation of the precipitates in the microstructure. Figure 1 illustrates the general framework of the multi-scale approach, encompassing the sub-grain scale with an explicit representation of the morphology of the $\gamma^{\prime }$ phase, the homogenized single crystal scale with parametric constitutive equations, and the polycrystalline grain scale comprising various grains. The sub-grain scale involves detailed dislocation mechanisms dependent on critical microstructural and mechanical features in plasticity models. Microstructural features encompass the morphology and crystallography of materials, while mechanical features include load intensity, load rate, and temperature.

In the realm of single crystals of superalloys, two distinct length scales can be delineated. The smaller scale, known as the sub-grain scale, involves an explicit representation of the γ-$\gamma^{\prime }$ phases, where the larger scale, or the homogenized grain scale, has implicit effects of morphology through parametric constitutive equations [27,28]. Two constitutive models are proposed for each scale, tailored to address dislocation mechanisms occurring either below or above the peak temperature, typically around 850 °C, where the maximum flow stress is observed. These models correspond to distinct dislocation mechanisms operative within their respective temperature ranges. At the sub-grain scale, the constitutive models are physics-based or dislocation density-based. For temperatures below the peak temperature, the constitutive model incorporates non-Schmid components to account for cross-slip mechanics. Conversely, for temperatures exceeding the peak temperature, the model employs the glide–climb creep model [29]. At the homogenized grain scale, the constitutive models take into account the implicit effects of both morphology and composition as constitutive parameters. The proposed constitutive model at this scale demonstrates the capability of encompassing temperature variations ranging from room temperature to 900 °C for various crystal orientations. Crystal plasticity models are employed in a hierarchical manner to integrate information at each scale, facilitating the implementation of the proposed constitutive models. This approach proves valuable for establishing microstructure–property relations and guiding microstructure design. This study focuses on developing a multi-scale model for cobalt-based superalloys to predict mechanical responses, utilizing a homogenized grain-scale crystal plasticity model suitable for design purposes. The method offers a significant efficiency advantage, wherein parametric hardening variables related to slip system resistances are expressed in terms of the γ-$\gamma^{\prime }$ morphology and composition. The overall model framework spans from the sub-grain γ-$\gamma^{\prime }$ level to the homogenized polycrystalline scale, which includes homogenized grains with an implicit presence of microstructural features.

## 3. Materials and Methods

**Figure 2:**
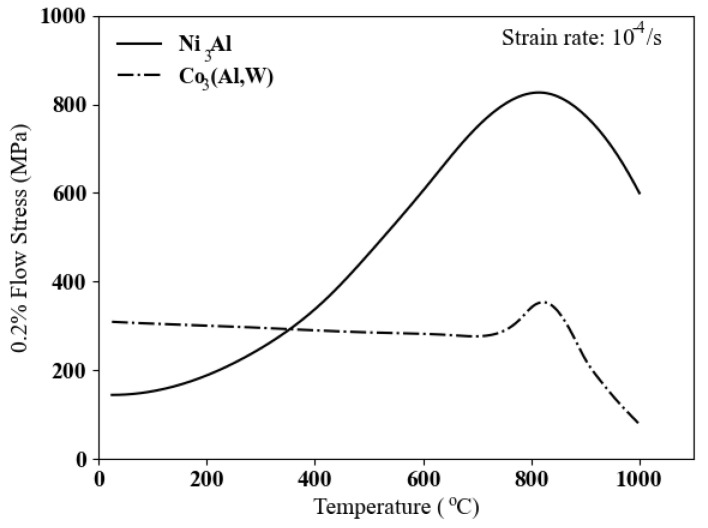
Stress anomalies in $\gamma^{\prime }$ phase of cobalt-based superalloys [14] and nickel-based superalloys [32].

The mechanical properties involving plastic deformation have been studied for both $\gamma^{\prime }$ precipitates [30] and two-phase γ-$\gamma^{\prime }$ [14,31]. Single crystals of ternary Co–Al–W-based alloys exhibit poorer mechanical properties compared to single crystals of nickel-based superalloys. However, the addition of elements such as Ta and Ti shows promise in enhancing high-temperature strength [14,31]. The mechanical properties of the ${\mathrm{Ni}}_{3}\mathrm{Al}$ compound in nickel-based superalloys exhibit anomalous behaviors from room temperature up to the peak temperature around 750 °C. This leads to a substantial increase in flow stress. In contrast, this region in $\gamma^{\prime }$ in cobalt-based superalloys is confined to a narrow temperature range between 650 °C to 850 °C, as illustrated in Figure 2. Furthermore, this has minimal effects on the flow stress for ternary Co–Al–W.

### 3.1. Constitutive Models for the Sub-Grain Level

The constitutive models at the sub-grain level are formulated using large deformation crystal plasticity. This involves a multiplicative decomposition of $F=F^{e}F^{p}$ where the total deformation gradient F is composed of an inelastic, incompressible part $F^{p}$, associated with slip without rotation, and an elastic part $F^{e}$, which accommodates rigid-body rotations and elastic stretching. For plastic velocity gradient $L^{p}$, the plastic shear strain rate $\dot{\gamma^{\alpha }}$ on the slip system α (including the slip direction $m_{0}^{\alpha }$ and slip plane normal $n_{0}^{\alpha }$ in the reference configuration) and the Schmid tensor $s_{0}^{\alpha }=m_{0}^{\alpha }⊗n_{0}^{\alpha }$ can be employed to calculate the evolution of plastic deformation as

(1) $$L^{p}={\dot{F}}^{p}F^{-p}=\sum_{\alpha =1}^{N}{\dot{\gamma }}^{\alpha }s_{0}^{\alpha }=\sum_{\alpha =1}^{N}{\dot{\gamma }}^{\alpha }m_{0}^{\alpha }⊗n_{0}^{\alpha }$$

The stress–strain relation invokes the second Piola–Kirchoff stress S and its work conjugate, Green–Lagrange strain tensor $E^{e}$, in the intermediate configuration

(2) $$S=\det\left(F^{e}\right)F^{e^{-1}}\sigma F^{e^{-T}}=C:E^{e}\;\;\;\mathrm{and}\;\;\;\;E^{e}=\frac{1}{2}\left(F^{e^{T}}F^{e}-I\right)$$

where C is a fourth order anisotropic elasticity tensor, σ the Cauchy stress tensor, and I the identity tensor.

The constitutive model utilized at the sub-grain scale draws inspiration from [13], wherein the plastic shear strain rate on a slip system adheres to the Orowan equation, ${\dot{\gamma }}^{\alpha }=\rho_{m}^{\alpha }b^{\alpha }v^{\alpha }$, which is contingent upon the mobile dislocation density, $\rho_{m}^{\alpha }$, and the Burgers vector, $b^{\alpha }$. The average velocity of the dislocations, influenced by the parallel and perpendicular slip resistances, $\tau_{\mathrm{pass}}^{\alpha }$ and $\tau_{\mathrm{cut}}^{\alpha }$, respectively, can be represented by this equation.

(3) $$\begin{array}{cc} v^{\alpha } & =v_{0}\exp\left(-\frac{Q}{K_{B}T}\right)\sinh{\left(\frac{|\tau^{\alpha }|\;-\;\tau_{\mathrm{pass}}^{\alpha }}{\tau_{\mathrm{cut}}^{\alpha }}\right)}^{p}\mathrm{sign}\left(\tau^{\alpha }\right). \end{array}$$

And with the hardening parameters as

(4) $$\begin{array}{cc} \tau_{\mathrm{pass}}^{\alpha } & =c_{2}Gb\sqrt{\rho_{P}^{\alpha }+\rho_{F}^{\alpha }},\;\;\;\tau_{\mathrm{cut}}^{\alpha }=\frac{c_{3}K_{B}T}{b^{2}}\sqrt{\rho_{F}^{\alpha }}, \end{array}$$

where *G* is the shear modulus.

The hardening parameters for passing and cutting are determined by the densities of parallel, $\rho_{P}$, and forest, $\rho_{F}$, dislocations, which are dislocation densities projected along and orthogonal to the slip planes. Statistically stored dislocations (SSDs), which are scalar and rate-dependent, contribute to the hardening through dislocation generation and annihilation processes. Additionally, due to the presence of plastic deformation gradients, particularly at the γ-$\gamma^{\prime }$ interface where misfit is a concern, slip resistance is further influenced by geometrically necessary dislocations (GNDs) [28]. GNDs are included to address this phenomenon, particularly noticeable at the γ-$\gamma^{\prime }$ boundaries. These superalloys exhibit strain gradient effects due to the dimensions of $\gamma^{\prime }$, which are captured by GNDs through morphological parameters such as shape, average size, and volume fraction of $\gamma^{\prime }$. A higher volume fraction for the same average size leads to more precipitates within a unit cell, resulting in a larger gradient of plastic deformation. Conversely, a larger average size for the same volume fraction increases channel width, thereby reducing the gradient of plastic deformation. As a result, the densities of parallel and forest dislocations can be expressed in terms of scalar SSDs and vectorial GNDs, influencing the mobile dislocation density $\rho_{m}^{\alpha }$ as described [27].

(5) $$\begin{array}{cc} \rho_{m}^{\alpha } & =\frac{c_{9}K_{B}T\sqrt{\rho_{F}^{\alpha }\rho_{P}^{\alpha }}}{Gb^{3}}. \end{array}$$

### 3.2. Constitutive Models for the Grain Level

**Figure 3:**
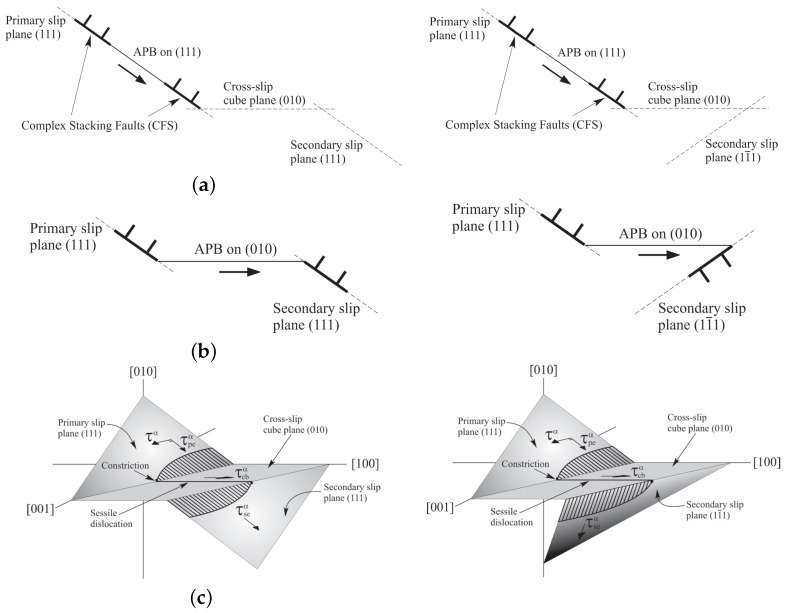
Kear–Wilsdorf lock formation: (**a**) Formation of anti-phase boundary. (**b**) Possible anti-phase boundary configurations. (**c**) Three-dimensional configuration of Kear–Wilsdorf lock.

There are two primary dislocation mechanisms observed from room temperature up to the peak temperature, which corresponds to the maximum flow stress at around 850 °C, and temperatures higher than that. Before reaching the peak temperature, the activation of octahedral slip systems is reported in both phases. As the temperature increases, anomalies in flow stress are observed, as depicted in Figure 2. These anomalies are attributed to dislocation behaviors in the intermetallic $\gamma^{\prime }$ phase, where screw dislocations tend to become locked in a Kear–Wilsdorf (KW) configuration, as illustrated in Figure 3, due to cross-slip [9]. The rate of lock formation rapidly increases as the temperature rises, reaching a maximum around the peak temperature.

**Figure 4:**
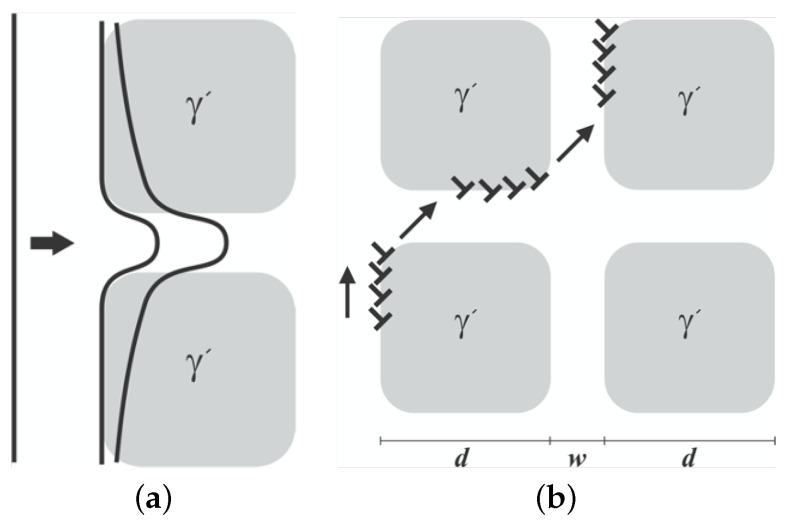
Glide and climb dislocation mechanisms: (**a**) Shearing. $\gamma^{\prime }$ (**b**) Climb ↑ and glide ↗.

At low to mid-temperatures, particularly at larger strains, dislocations in the channel tend to shear $\gamma^{\prime }$ precipitates, and this rate increases with rising temperature. Similar to nickel-based superalloys, superdislocations have been observed instead of regular dislocations in cobalt-based superalloys [14]. These superdislocations exhibit a behavior where they change planes and cross-slip along the anti-phase boundary (APB). However, at elevated temperatures, the dislocations lack sufficient energy to cut through the precipitates. Instead, they glide just inside the channel of the γ phase [14,33,34]. This glide in the channel typically continues by climbing along the precipitates, as illustrated in Figure 4, giving rise to a glide–climb dislocation mechanism [27,29].

Accordingly, two constitutive models are employed to address the distinct dislocation mechanisms. The first model accommodates the cross-slip mechanism, primarily occurring at low to mid-temperatures. The second model addresses the glide–climb mechanism, predominant at higher temperatures.

#### 3.2.1. Constitutive Model for Cross-Slip Mechanism

The grain level, or homogenized single crystal grain-scale, for cobalt-based superalloys is obtained from a parametrized representation of the morphology of the precipitates, following the method explained in [35]. This model is a function of the microstructure’s morphology, especially the precipitates, containing their shape, size, and volume fraction of the $\gamma^{\prime }$ phase. The homogenized constitutive model is an Orowan-based model utilizing hardening parameters of thermal shear resistance, $\tau_{*}^{\alpha }$, and athermal or passing shear resistance, $\tau_{pass}^{\alpha }$, which reflect the effects of forest and parallel dislocation densities, as employed in the sub-grain scale, stated as

(6) $${\dot{\gamma }}^{\alpha }=\left\{\begin{array}{cc} 0 & \mathrm{for}\;\tau_{\mathrm{eff}}^{\alpha }\leq 0\\ {\dot{\gamma }}_{*}^{\alpha }\exp\left\{-\frac{Q}{K_{B}\theta }{\left[1-{\left(\frac{|\tau_{\mathrm{eff}}^{\alpha }|}{\tau_{*}^{\alpha }}\right)}^{p}\right]}^{q}\right\}\mathrm{sign}\left(\tau^{\alpha }\right) & \mathrm{for}\;0<\tau_{\mathrm{eff}}^{\alpha }\leq \tau_{*}^{\alpha } \end{array}\right.$$

Dislocations become active when the effective shear stress, represented by the difference between the resolved shear stress, $\tau^{\alpha }$, and the passing stress, $\tau_{pass}^{\alpha }$, are expressed as $\tau_{eff}^{\alpha }=\left|\tau_{\alpha }-\tau_{pass}^{\alpha }\right|$, which is greater than zero. This implies that the resolved shear stress on a slip system must exceed the passing stress for dislocation motions to occur. In general, $\tau_{*}^{\alpha }$ represents the cutting stress, $\tau_{*}^{\alpha }=\tau_{cut}^{\alpha }$, as a morphology-dependent parameter in the homogenized model [13], though additional terms are needed to be modified for the cross-slip mechanism and also to account for the composition effects. The reference shear strain rate, ${\dot{\gamma }}_{*}^{\alpha }$, is a function of plastic strain and morphological parameters [35].

Both cutting and passing shear resistances undergo evolution due to self and latent hardening, and this evolution is defined as

(7) $${\dot{\tau }}_{pass}^{\alpha }=\sum_{\beta =1}^{N}h_{a}^{\alpha \beta }|{\dot{\gamma }}^{\beta }sin\left(n^{\alpha },t^{\beta }\right)|,\;\;\;{\dot{\tau }}_{cut}^{\alpha }=\sum_{\beta =1}^{N}h_{*}^{\alpha \beta }\left|{\dot{\gamma }}^{\beta }cos\left(n^{\alpha },t^{\beta }\right)\right|$$

The total slip resistance rate can be written as ${\dot{s}}^{\alpha }=\sqrt{{\left({\dot{\tau }}_{pass}^{\alpha }\right)}^{2}+{\left({\dot{\tau }}_{*}^{\alpha }\right)}^{2}}$. The hardening matrix, $h^{\alpha \beta }$, can be calculated using the interaction matrix, which accounts for interactions between slip systems by

(8) $$h^{\alpha \beta }=q^{\alpha \beta }h^{\beta },\;\;\;\mathrm{where}\;\;h^{\beta }=\left[h_{0}{\left(1-\frac{s^{\beta }}{s_{sat}^{\beta }}\right)}^{r}\right]sign\left(1-\frac{s^{\beta }}{s_{sat}^{\beta }}\right)$$

The resistance parameter, $h^{\beta }$, is a function of the saturation shear resistance, $s_{sat}^{\beta }$, and total slip system resistance. $h_{0}$ and *r* are material constants, and $q^{\alpha \beta }=q+\left(1-q\right)\delta^{\alpha \beta }$ is the interaction coefficient matrix, which includes *q* as a latent-hardening factor taken to be 1.4.

#### 3.2.2. Modification of $\tau_{*}^{\alpha }$ for KW Lock Formation Due to Cross-Slip Mechanism

As mentioned in the previous section, an additional term is required to integrate into the thermal shear resistance, $\tau_{*}^{\alpha }$, to accommodate cross-slip resistance resulting in $\tau_{*}^{\alpha }=\tau_{cut}^{\alpha }+\tau_{\mathrm{cross}}^{\alpha }$. Locked cross-slipped dislocations, as depicted in Figure 3, increase the hardening by acting as obstacles to further dislocation motions. The enthalpy term for the cross-slip mechanism, developed in [13] for nickel-based superalloys, is used and modified for cobalt-based superalloys. In this modification, the non-Schmid components of $\tau_{pe}^{\alpha },\tau_{se}^{\alpha }$, and $\tau_{cb}^{\alpha }$ are responsible for dislocation dissociation during the cross-slip mechanism. However, there are no dislocation core effects for cube slip systems. Therefore, the cross-slip resistances are considered only for the octahedral slip systems as a function of non-Schmid factors and temperatures. For cube slip systems, it will be solely a function of temperature.

(9) $$\tau_{\mathrm{cross}}^{\alpha }=\left\{\begin{array}{cc} \tau_{\mathrm{crossco}}^{\alpha }=\tau_{\mathrm{crossco}}^{\alpha }\left(\tau_{\mathrm{pe}}^{\alpha },\tau_{\mathrm{se}}^{\alpha },\tau_{\mathrm{cb}}^{\alpha },\theta ,\Gamma_{111},\Gamma_{010}\right) & \mathrm{on}\;\mathrm{octahedral}\;\mathrm{slip}\;\mathrm{systems}\\ \tau_{\mathrm{crosscc}}=\tau_{\mathrm{crosscc}}\left(\theta \right) & \mathrm{on}\;\mathrm{cube}\;\mathrm{slip}\;\mathrm{systems} \end{array}\right.$$

Following [13], the cross-slip shear resistance can be stated as

(10) $$\begin{array}{c} \tau_{\mathrm{crossco}}^{\alpha }=\xi_{0}exp\left(\frac{A}{\theta -\theta_{c}}\right)G\sqrt{\rho_{0}\exp\left(-\frac{H^{\alpha }}{K_{B}\theta }\right)}\\ \;\;\;\;\;\;\;\;\;\;\;\;\mathrm{where}\;\;H^{\alpha }=c_{H}\left\{h+k_{1}\left(t_{\mathrm{pe}}^{\alpha }-k_{1}t_{\mathrm{se}}^{\alpha }\right)+\sqrt{\left(\frac{1}{\sqrt{3}}-\frac{\Gamma^{010}}{\Gamma^{111}}+\left|t_{\mathrm{cb}}^{\alpha }\right|\right)\frac{b}{B}}\right\} \end{array}$$

The temperature parameter $\xi =\xi_{0}exp\left(\frac{A}{\theta -\theta_{c}}\right)$ signifies the strength of the pinning obstacles and diminishes as temperature rises. The rate of decrease for this parameter accelerates notably as the upper boundary temperature $\theta_{c}$ is approached, at which climb mechanism ensues. Initially, this parameter has a value exceeding unity for temperatures below the peak temperature, but it decreases to less than unity for temperatures beyond that. The yield stresses corresponding to three temperatures of 300 K, 900 K, and 1200 K are utilized to calibrate the three parameters $\xi_{0}$, *A*, and $\theta_{c}$, which results in 5.5, 50, and 880, respectively. G is the shear modulus, and $\rho_{0}$ is the initial cross-slip dislocation density set to be $5.0*10^{15}$. The enthalpy term has material constants $k_{1}$ and $k_{2}$ calibrated as 0.2, and 0.7, $\Gamma^{111}$, and $\Gamma^{010}$ (${J/m}^{2}$) are the APB energies per unit area on the octahedral and cube planes, respectively. $t_{xx}^{\alpha }$ is the normalized non-Schmid component of the resolved shear stress as $t_{xx}^{\alpha }=\frac{\tau_{xx}^{\alpha }}{\Gamma^{111}/b}$, $B=\frac{Gb^{2}}{2\pi \Gamma^{111}}$, and $c_{H}=\frac{Gb^{3}}{4\pi }$. The cross-slip shear stress increases as the dislocation densities of the cross-slip mechanism rise. Conversely, the obstacle strength diminishes with rising temperature. Therefore, there is a competition between the increasing strength with the formation of KW locks and the decreasing obstacle strength with increasing temperature.

### 3.3. Homogenized Constitutive Model from the Sub-Grain Representative Volume Element (RVE) Model

The constitutive parameters described in Equation (6) for the homogenized single crystal grain-scale model are influenced by macro–micro energy equivalence. In this framework, the micromechanical analysis is carried out using the sub-grain RVE model. Functional parameters within the constitutive model are expressed in relation to morphological variables.

The sub-grain microstructural RVE comprises a two-phase γ-$\gamma^{\prime }$ system, characterized by three morphology variables to account for the volume fraction, shape, and size of the $\gamma^{\prime }$ phase. These variables are designated as follows: (1) $V_{p}=\frac{V_{\gamma^{\prime }}}{V_{RVE}}$, for volume fraction of the $\gamma^{\prime }$ phase; (2) *n*, for the shape factor of the $\gamma^{\prime }$ phase, which is obtained by considering $\gamma^{\prime }$ as a super-ellipsoid with the same dimension in principle directions a=b=c in the equation ${\left(\frac{x}{a}\right)}^{n}+{\left(\frac{y}{b}\right)}^{n}+{\left(\frac{z}{c}\right)}^{n}=1$; (3) *w*, the distance between precipitates.

The constitutive parameters at the single crystal grain scale are denoted as $\tau_{cut0}^{\alpha }\left(V_{p},n,w\right)$, $\tau_{cross}^{\alpha }\left(V_{p},n,w\right)$, and $s_{sat}^{\alpha }\left(V_{p},n,w\right)$, as expressed in Equation (6) by comparing the stress–strain response obtained from the homogenized constitutive model with the volume-averaged response from simulations of the sub-grain RVE model. This calibration process involves generating various RVE microstructures with different morphologies, including distinct volume fractions $\left(V_{p}\right)$, shape factor (n), and channel width (w) [35]. Subsequently, dislocation density-based models at the sub-grain scale are applied to each of these microstructures to simulate volumetric stress–strain curves up to 10% true strain. To adhere to the Hill–Mandel principle, the same volumetric stress–strain response is replicated at the grain scale using the homogenized constitutive model by adjusting four constitutive parameters. This comprehensive set of simulations yields the functional forms of the single crystal constitutive parameters through the least square minimization method. The final form of these constitutive parameters can be found in [28]. Ultimately, the homogenized single crystal grain-scale model with parametric constitutive relations is validated against findings from the sub-grain scale and experimental data, as documented in [13]. This validation demonstrates a significant acceleration in the processing speed by approximately four orders of magnitude, all while maintaining a high level of accuracy.

### 3.4. Modification of $\tau_{*}^{\alpha }$ for the Effect of Composition

Experimental data indicate that the addition of certain elements, such as Ta and Ti [7,8,14], has positive effects on mechanical properties, including yield stress and creep resistance. Yet, the inclusion of materials like Cr can have negative effects on these mechanical outcomes. In other words, the addition of these materials alters the slip system resistances. Generally, they can be incorporated into the total slip system resistance as

(11) $$\begin{array}{cc} \; & \tau_{\mathrm{comp}}^{\alpha }=\sum_{m}x_{m}\tau_{m}^{\alpha }\left(T\right),\;\;\;\;\left(m=Ta,Ti,Cr\right) \end{array}$$

where $x_{m}$ is the atomic concentration of element m and $\tau_{m}^{\alpha }$ is the temperature-dependent shear resistance to account for compositions.

Now, the total slip system resistance $\tau_{*}^{\alpha }$ can be stated as

(12) $$\begin{array}{c} \tau_{*}^{\alpha }=\tau_{cut}^{\alpha }+\tau_{cross}^{\alpha }+\tau_{comp}^{\alpha } \end{array}$$

### 3.5. Constitutive Model for Climb and Glide Mechanism

At elevated temperatures and under lower applied stress, dislocations are unable to cut through the precipitates. Instead, they climb along the interface of γ-$\gamma^{\prime }$ towards the matrix, where glide takes place in the channel before reaching the next precipitates [29], as illustrated in Figure 4b.

The rate of plastic shear strain for a given slip system can be calculated in terms of the morphology of γ-$\gamma^{\prime }$ and the composition of γ-$\gamma^{\prime }$ using the Orowan-based constitutive model, as outlined in [27] by

(13) $$\begin{array}{c} {\dot{\gamma }}^{\alpha }=2\rho_{m}^{\alpha }V_{p}\left(1-V_{p}\right)b\frac{w}{d}\Gamma_{e}^{\alpha } \end{array}$$

$V_{p}$, *d*, and *w* represent the γ-$\gamma^{\prime }$ morphology volume fraction, average size, and average distance between the precipitates, respectively, while $\Gamma_{e}^{\alpha }$ is the released frequency containing the effects of composition and $\rho_{m}^{\alpha }$ is the mobile dislocation density. Utilizing Gibbs free energy [36], the released frequency can be rewritten as

(14) $$\begin{array}{c} \Gamma_{e}^{\alpha }=D_{eff}^{\alpha }{\left[1-{\left(\frac{\tau_{eff}^{\alpha }}{s^{\alpha }}\right)}^{p}\right]}^{q} \end{array}$$

where the effective diffusion coefficient due to the climb mechanism is

(15) $$\begin{array}{c} D_{eff}^{\alpha }={\dot{\gamma }}_{0}^{\alpha }\exp\left\{-\frac{Q^{\alpha }}{K_{B}T}\right\} \end{array}$$

Hence, the crystal plasticity constitutive model for the climb and glide mechanism can be stated with

(16) $$\begin{array}{c} {\dot{\gamma }}^{\alpha }=2\rho_{m}^{\alpha }V_{p}\left(1-V_{p}\right)D_{eff}^{\alpha }\left(\frac{w}{bd}\right)\exp\left[1-\left(\frac{\tau_{eff}^{\alpha }}{\tau_{*}^{\alpha }}\right)\right] \end{array}$$

The effective diffusivity in Equation (16) is considered by the Arrhenius representation of $D_{eff}^{\alpha }=D_{eff0}^{\alpha }\exp\left(-\frac{Q_{eff}^{\alpha }}{K_{B}T}\right)$, where $D_{eff0}^{\alpha }$ and $Q_{eff}^{\alpha }$ are the effective pre-exponential coefficient and activation energy, respectively.

The effective activation energy is assessed in terms of the activation energy [29] of the constituents in the composition. This assumes that the impact of solute elements on the solvent alters the effective activation energy in pure cobalt, considering an average weighted percentage of the various solutes by

(17) $$\begin{array}{c} Q_{eff}^{\alpha }=Q_{Co}^{\alpha }+\sum_{m}x_{m}Q_{m,Co}^{\alpha } \end{array}$$

where $Q_{Co}^{\alpha }$ is the activation energy for self-diffusion of cobalt and $Q_{m,Co}^{\alpha }$ the activation energy for the solute with the atomic concentration of *m*. The effective pre-exponential coefficient can be calculated in terms of different elements in the composition and the weighted percentage; $D_{eff0a}^{\alpha }=\sum_{m}x_{m}D_{0mCoi}^{\alpha }$, where $D_{0m,Co}^{\alpha }$ is the pre-exponential factor of solute concentration in cobalt.

## 4. Results and Discussion

### 4.1. Validation of the Computational Model with Experiments

**Figure 5:**
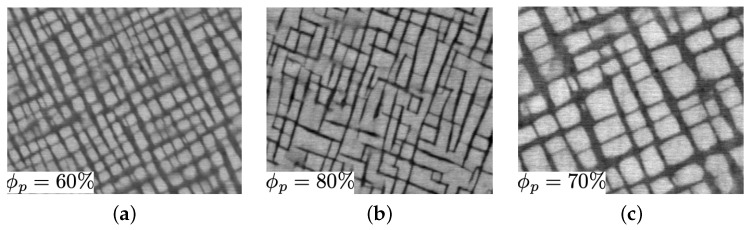
Cobalt-based superalloy microstructures: (**a**) Co–9Al–9W, (**b**) Co–9Al–11W, (**c**) Co–9Al–9W–2Ta [14].

The computational model at the grain scale for cobalt-based superalloys incorporates two dislocation mechanisms over a temperature range from 25 °C to 1000 °C. At lower temperatures up to 800 °C, the yield stress experiences almost a linear decline from room temperature to around 600 °C, followed by an anomaly between 600 °C to 800 °C. This anomaly is primarily attributed to the cross-slip mechanism following KW locking formations. At temperatures higher than 800 °C, a sharp decline is observed, indicating that dislocations do not have enough energy to cut through the precipitates; instead, they glide inside the channel and climb along the precipitates. To validate both constitutive models, yield stresses from room temperature up to 1000 °C are computed and compared to the experimental data. Hence, simulations were conducted for three ternaries with the nominal chemical compositions (mole fraction × 100) of Co–9.2Al–9W, referred to as 9 W; Co–9.4Al–10.7 W, referred to as 11 W; and Co–8.8Al–9.8W–2Ta, referred to as 2 Ta. The microstructures after heat treatments are shown in Figure 5. According to [14], the microstructures were homogenized at temperatures between their $\gamma^{\prime }$ solvus (around 1300 K) and solidus (around 1700 K) temperatures, and then aged at temperatures about 60 K to 130 K below the $\gamma^{\prime }$ solvus temperatures. Subsequently, the simulation results were compared with the experimental data reported in [14]. The volume fraction in 9 W is 60%, which is increased by adding tungsten content in 11 W to 80% and in 2 Ta to 70%.

**Figure 6:**
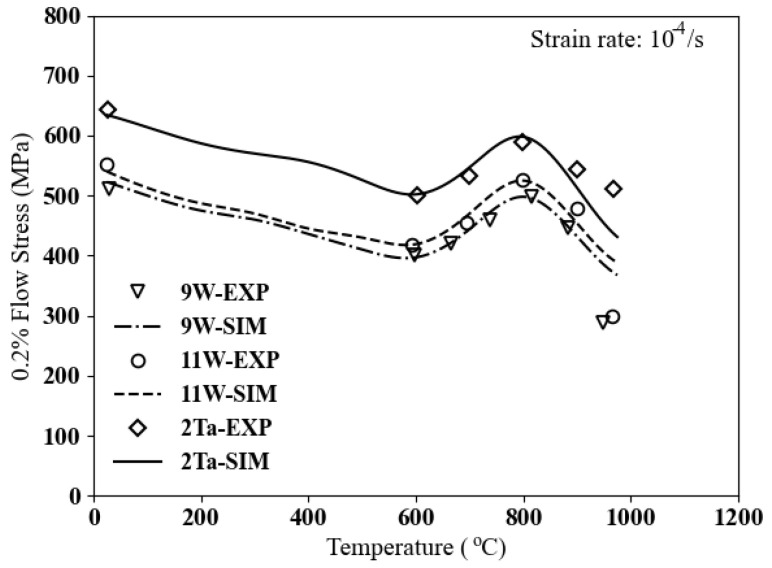
Comparison of yield stress vs. temperature between simulations and experimental data for cobalt-based superalloys 9 W, 11 W, and 2 Ta [14].

The variation of 0.2% flow stress vs. temperature is illustrated in Figure 6. All tests depicted in Figure 6 entail tensile tests conducted under a constant strain rate of $10^{-4}$ 1/s. The volume fraction of precipitates varies: 60% for Co–9Al–9W, 80% for Co–9Al–11W, and 70% for Co–9Al–9W–2Ta. According to the data, the changes in flow stress due to the addition of tungsten content from 9 W to 11 W are negligible. However, adding 2% Ta results in an increase in flow stress of almost 20% for temperatures below 800 °C. The deviation between experimental and computational data at very high temperatures may be related to the rafting mechanism, which is not considered in this study.

#### 4.1.1. Composition Effects on Flow Stress

Experimental data indicate that certain elements, such as tungsten, tantalum, and titanium, have positive effects on mechanical properties, including flow stress and creep resistances, whereas elements like chromium exhibit negative effects on these properties. The impact of these elements depends on the testing temperature, with the positive or negative effects diminishing as the temperature increases. These effects can be expressed in Equation (11) as a modification to the slip system resistances. In this equation, $\tau_{m}^{\alpha }$ will have a general form, particularly under constant strain rate by

(18) $$\begin{array}{c} \tau_{m}^{\alpha }=\tau_{m0}*exp\left(\frac{T_{ref}-T}{1000}\right) \end{array}$$

$\tau_{m0}^{\alpha }$ is the initial shear resistance, which is positive for Ta and Ti and negative for Cr. $T_{ref}$ is the reference temperature, which is calibrated to be 1100 Kelvin for all three elements. The equation is valid for the temperatures under $T_{ref}$. The initial shear resistance along with the reference temperature is provided in Table 1.

**Figure 7:**
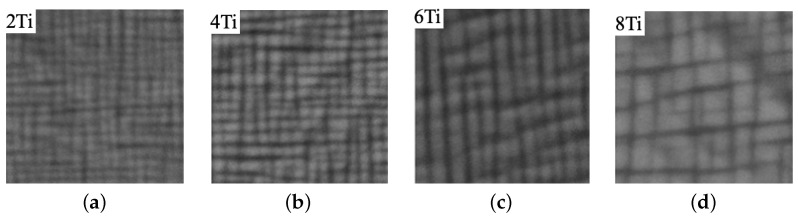
Cobalt-based superalloy microstructure aged at 1173 K for 168 h, Co-10Ni-(9 − x)Al–(9 − x)W–2xTi: (**a**) 2Ti, (**b**) 4Ti, (**c**) 6Ti, (**d**) 8Ti [37].

To investigate the effects of elements such as tungsten, tantalum, and titanium on flow stress, the microstructures provided in [37] are considered. The base microstructure has a nominal chemical composition (mole fraction × 100) of Co–10Ni–(9 − x)Al–(9 − x)W–2xTi. The flow stress at different temperatures is similar to the ternary case when x is 0. The microstructures for x = 1 to 4 are shown in Figure 7 as 2 Ti, 4 Ti, 6 Ti, and 8 Ti. According to [37], the volume fraction of the precipitates increases from 56% to 68% when the content of titanium in mole fraction × 100 rises from 0 to 8%.

**Figure 8:**
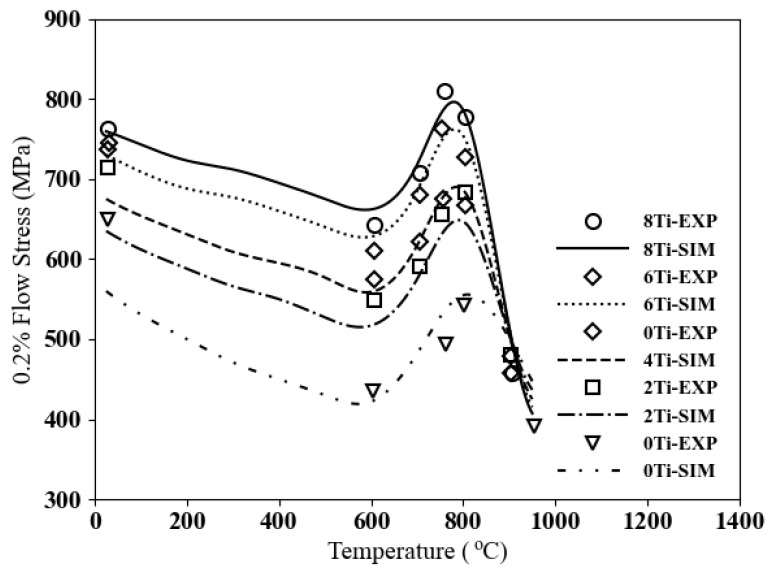
Yield stress vs. temperature for cobalt-based superalloys Co–10Ni–(9 − x)Al–(9 − x)W–2xTi compared to the experimental data [37].

The variation of flow stresses with respect to temperature for these five microstructures is depicted in Figure 8. It is evident that adding titanium, even just 2%, increases the yield stress by more than 20% at the peak temperature. All microstructures with titanium content exhibit an increase in flow stress at temperatures below 900 °C. The flow stress for 8 Ti shows competitive values with nickel-based superalloys.

#### 4.1.2. Validation of the Creep Model with Experiments

**Figure 9:**
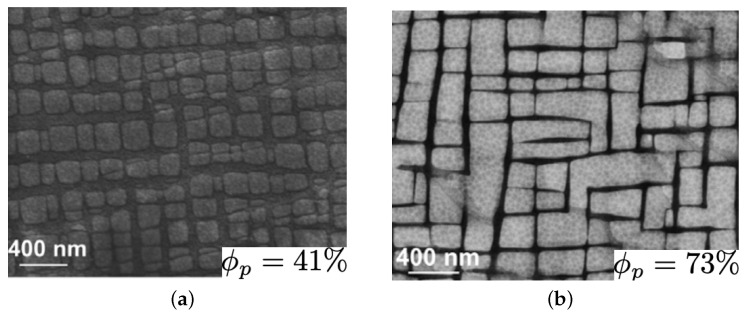
Cobalt-based superalloy ternary microstructure with different contents of Tungsten: (**a**) Co–9Al–9W, (**b**) Co–9Al–11W [34].

At elevated temperatures, gliding inside the channel and climbing along precipitates will be the dominant mechanism. The plastic shear strain or creep properties can be measured using Equation (16). The first study will be on the creep behaviors of ternary cobalt-based superalloys and the effect of tungsten content. As learned from Figure 6, tungsten will have small effects on flow stress, which is the onset of plastic strain. To understand the effect of W on the creep properties, two ternary microstructures are taken from [34], as shown in Figure 9, with the composition of Co–9Al–9W and Co–9Al–11W having 41% and 73% of the $\gamma^{\prime }$ volume fraction, respectively.

**Figure 10:**
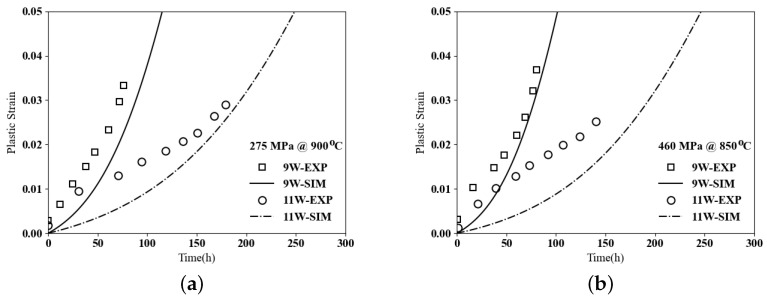
Creep curves, plastic strain vs. time for ternary Co–9Al–xW (**a**) Under 275 MPa at 900 °C. (**b**) Under 460 MPa at 850 °C.

Creep tests on each microstructure are conducted, with the first test under 275 MPa at 900 °C and the second one under 460 MPa at 850 °C. The experimental data are compared with the computational results in Figure 10. As noted, adding more tungsten increases the creep resistance in cobalt-based superalloys, which can be explained by two reasons. First, tungsten is a slow diffuser, and second, adding more W increases the volume fraction of $\gamma^{\prime }$, which is the main morphology factor strengthening creep properties in cobalt-based superalloys.

**Figure 11:**
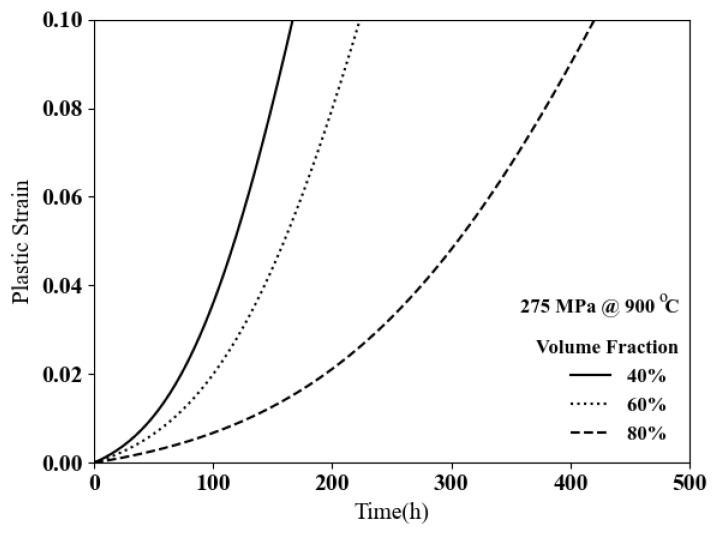
Creep resistances for different volume fractions of the $\gamma^{\prime }$ phase in ternary cobalt-based superalloys.

To investigate the effect of volume fraction on creep responses, three simulations are performed for tests with the applied stress of 275 MPa at 900 °C based on the constitutive model stated in Equation (16). The volume fraction of $\gamma^{\prime }$ in ternary cobalt-based superalloys can be changed by altering the content of tungsten. The change in volume fraction of $\gamma^{\prime }$ from 40% to 80% will significantly decrease the plastic strain, as shown in Figure 11. The plastic strain in the microstructure with 40% of the $\gamma^{\prime }$ volume fraction will be more than 10% in less than 200 h, while the plastic strain in the microstructure with 80% of the $\gamma^{\prime }$ volume fraction will show less than 2% plastic strain.

#### 4.1.3. Effects of Composition on Creep Properties in Cobalt-Based Superalloys

**Figure 12:**
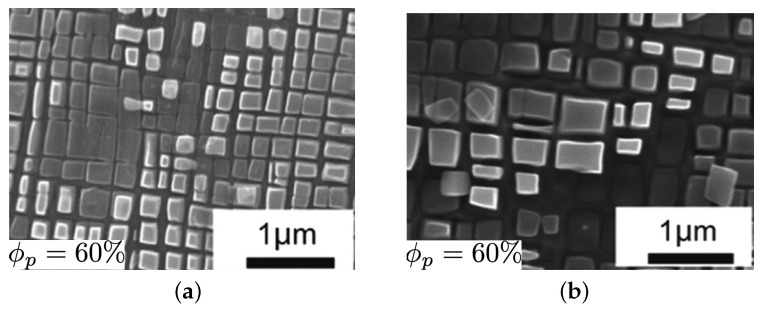
Cobalt-based superalloy ternary microstructure with different contents of tantalum, Co–9.5Al–5W–16Ni–6Cr–xTa (**a**) 1.8 Ta with the average size of 241 µm. (**b**) 2.8 Ta with the average size of 301 µm [38].

Composition has significant effects on the creep properties of cobalt-based superalloys. In this section, we inspect the effects of Ti, Ta, and Cr and combinations of these elements in the composition on creep properties. The first composition is Co–9.5Al–5W–16Ni–6Cr–xTa, reported by [38]. The microstructures with 1.8 Ta and 2.8 Ta are taken from [38] and shown in Figure 12.

**Figure 13:**
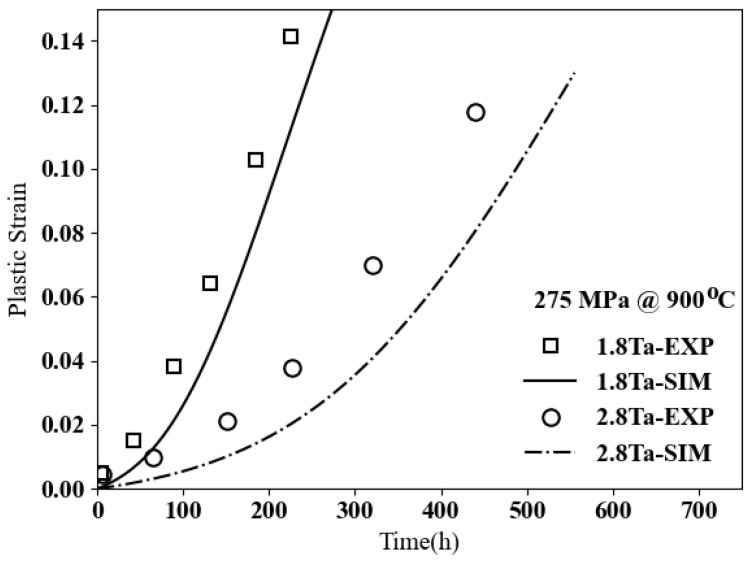
Creep resistances for different contents of tantalum in cobalt-based superalloys of Co–9.5Al–5W–16Ni–6Cr–xTa.

As reported in [38], increasing Ta from 1.8% to 2.8% has slight effects on the volume fraction of $\gamma^{\prime }$; however, it results in coarser precipitates, where the average size increases from 241 µm to 301 µm. The variation of the plastic deformation over time for these two microstructures under 275 MPa at 900 °C is shown in Figure 13. The results indicate that increasing Ta by 1% improves creep resistance notably, with the plastic strain for this composition being around 2% after 200 h, while for 1.8 Ta, it will be more than 10%. The simulation results and the experimental data have the same trend; however, there are some differences between them, which are possibly due to the high content of nickel in the compositions, which are not investigated in the current research.

**Figure 14:**
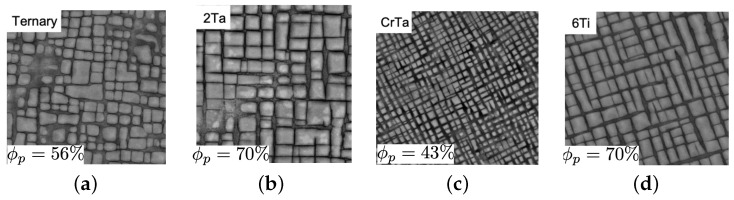
Cobalt-based superalloy microstructure: (**a**) Co–9Al–11W, (**b**) Co–9Al–10W–2Ta, (**c**) Co–8Al–8W–1.5Ta–4.5Cr, (**d**) Co–7Al–8W–6Ti [7].

The second example from [7] provides better insights into how these three elements in the composition will change the creep behaviors. The base microstructure is ternary cobalt-based superalloys Co–9Al–11W with a volume fraction of 56%, as shown in Figure 14. All microstructures are subjected to creep under 310 MPa at 900 °C.

**Figure 15:**
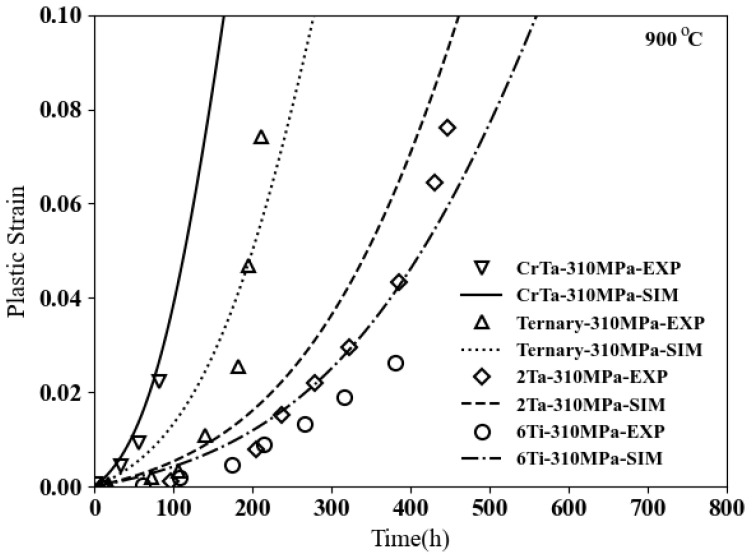
Creep resistances for different compositions of cobalt-based superalloys.

As shown in Figure 15, the plastic deformation for the ternary will be around 8% after 200 h. By adding 2% Ta to the ternary, the volume fraction will increase to 70%, as illustrated in Figure 14b, where the plastic strain will be around 1% after 200 h and less than 8% after 400 h for this microstructure, which exhibits the positive effects of adding tantalum. Now, if we add 4.5% Cr to the previous microstructure, the volume fraction will decrease to 43%, as demonstrated in Figure 14c; however, it diminishes the creep resistances dramatically, with the plastic deformation being more than 10% after 200 h. In fact, the creep strength for this microstructure is less than the ternary one. A total of 6% Ti is added to the ternary for the last tested microstructure, as shown in Figure 14d, to increase the volume fraction to 70%. The remarkable increase in creep resistance can be seen in Figure 15, where the plastic strain is less than 3% after 400 h. Based on the current experimental data obtained from the high content titanium samples, it seems that the simulation is providing lower predictions. With access to more experimental data, it would be possible to better evaluate the accuracy of the computational approach’s convergence.

## 5. Conclusions

A comprehensive computational framework is proposed to predict the mechanical properties of cobalt-based superalloys. The framework employs a multi-scale approach to capture the effects of both morphology and composition. Applicable from room temperature to 900 °C, the model accounts for two dominant dislocation mechanisms: at lower temperatures, precipitates are cut under sufficient loading, while at higher temperatures, they glide inside the channel and climb around precipitates. The homogenized constitutive models for simulating both mechanisms consider the effects of morphology and composition. The mechanical properties, such as flow stress and creep strength, in ternary cobalt-based superalloys can be notably improved by adding certain elements to the composition, making them competitive with nickel-based superalloys. Simulations indicate that increasing tungsten content significantly enhances the volume fraction of $\gamma^{\prime }$ and creep resistances, with minimal effects on flow stress. Additionally, adding tantalum or titanium, even in small amounts, has substantial increasing effects on both flow stress and creep resistance. The weakening effects of chromium on both flow stress and creep strength are also noted. Additionally, the model holds promise for simulating material behavior under extreme conditions, offering insights into its potential applications across various industrial settings. Exploring these avenues further could provide valuable insights into the practical utility of our model.

As part of future work, the constitutive models will be integrated into the Object-Oriented Finite Element (OOF) Framework developed by scientists at the National Institute of Standards and Technology (NIST). This publicly accessible platform aims to assist materials scientists and engineers in conducting computational investigations of structure–property relations, particularly in systems dominated by crystal plasticity in mechanical behavior.

## Figures and Tables

**Table 1:** Nominal chemical composition (mass fraction) for four generations of single-crystal nickel-based superalloys.

Element	Ta	Ti	Cr
$\tau_{m0}*10^{2}$ (MPa)	170	45	−17
$T_{ref}$ (K)	1100	1100	1100

## Data Availability

The data that support the findings of this study are available on request from the corresponding author.
